# Application and Optimization of Wavelet Transform Filter for North-Seeking Gyroscope Sensor Exposed to Vibration

**DOI:** 10.3390/s19163624

**Published:** 2019-08-20

**Authors:** Ji Ma, Zhiqiang Yang, Zhen Shi, Xuewei Zhang, Chenchen Liu

**Affiliations:** School of Geology Engineering and Geomatics, Chang’an University, Xi’an 710054, China

**Keywords:** north-seeking gyroscope, wavelet transform, filtering de-noising, magnetic levitation gyroscope (GAT), time frequency analysis

## Abstract

Conventional wavelet transform (WT) filters have less effect on de-noising and correction of a north-seeking gyroscope sensor exposed to vibration, since the optimal wavelet decomposed level for de-noising is difficult to determine. To solve this problem, this paper proposes an optimized WT filter which is suited to the magnetic levitation gyroscope (GAT). The proposed method was tested on an equivalent mock-up network of the tunnels associated with the Hong Kong‒Zhuhai‒Macau Bridge. The gyro-observed signals exposed to vibration were collected in our experiment, and the empirical values of the optimal wavelet decomposed levels (from 6 to 10) for observed signals were constrained and validated by the high-precision Global Navigation Satellite System (GNSS) network. The result shows that the lateral breakthrough error of the tunnel was reduced from 12.1 to 3.8 mm with a ratio of 68.7%, which suggests that the method is able to correct the abnormal signal of a north-seeking gyroscope sensor exposed to vibration.

## 1. Introduction

A gyro total station (gyrotheodolite) is a north-seeking instrument that combines a gyroscope sensor and total station (theodolite), which can measure true geographical azimuths in narrow spaces where satellite signals cannot be received [1,2]. The technology of the north-seeking gyroscope is widely used in the fields of breakthrough measurement of super-long tunnels, indoor positioning systems and quick orientation of mobile missile sites [3,4,5].

During orientation measurement in a tunnel, gyro data are not only affected by the constant and random drift errors of the sensor but also the external environmental factors, such as blasting, construction vibration, air draft-induced vibration of passing vehicles [6,7], magnetic field interference, and changes in temperature, humidity and air pressure [8,9]. These interference factors (especially physical vibration) cause abnormalities of the gyro signal, which contains significant non-stationary noise, leading to distortion of the orientation results [10].

The wavelet transform (WT) proposed by Morlet is a signal analysis and filtering method with high resolution in both the time and frequency domains [11], which has applicability in processing non-linear and non-stationary data. The basic idea is to use a window function to construct a basis function for orthogonal transformation [12]. The signal is decomposed into a series of wavelet coefficients, including high-frequency and low-frequency coefficients, by choosing an appropriate wavelet function. The high-frequency coefficients are limited according to the corresponding threshold criteria, and then reconstructed with low-frequency coefficients to achieve the purpose of de-noising and correction [13,14]. With the development of the WT filter, the application field has been greatly expanded to include biomedicine [15,16,17], geophysics [18,19], image processing [20,21], tracking detection [22,23,24], and feature extraction [25,26,27]. The wavelet filter also has precedents for signal processing of gyroscope sensors of the strapdown inertial navigation system (SINS) used for attitude determination, such as the fiber optic gyroscope (FOG) [28,29] and micro electro mechanical system (MEMS) gyroscopes [30,31], for which good application results have been achieved in previous studies [32,33,34,35]. However, the widely-used suspension tape gyroscope sensor is limited by the tracking north-seeking mode of collecting scattered points, which cannot easily simulate the changeable environment in real-time. Since there are fewer redundant observations of this north-seeking mode, the application effect of the filtering model is not significant [10].

An innovative new type of north-seeking gyroscope, the magnetic levitation gyroscope (GAT), developed by the Chang’an University and China Space Age Electronics 16th Research Institute in 2008, adopts a magnetic suspension-supporting and non-contact photoelectric technology to measure the true geographical azimuth (the nominal orientation accuracy is 3.5″ in 8 min) [36]. The GAT has the capacity to dynamically record large amounts of north-seeking parameters in real-time, which allows monitoring of a changing environment (40,000 groups of observed signals per orientation result). It is possible to optimize the non-stationary gyro data with modern filtering technology and extract the effective components from the noisy signals, thus improving the north-seeking orientation accuracy [37]. Since 2008, the GAT has been widely used in several major underground engineering projects in China and provided reliable orientation checks for the breakthrough of the tunnels, such as in the cases of the Qinghai–Tibet Railway Tunnel [38], the Hanjiang River Diversion to Weihe River Qinling Water Conveyance Tunnel [39], and the Immersed Tunnel of the Hong Kong-Zhuhai-Macao Bridge [10].

However, there are some problems in the application of the WT filter to GAT data correction and de-noising: (1) The filters applicable for FOG and MEMS have less effect on the application of the GAT since their observed signal distribution characteristics are different [10]. (2) Some researchers suggested that the optimal decomposed level is 5 for the gyro signal [40], while others considered that the decomposed level of 10 is a better choice [28,41,42], but the reasons for these choices were not given. Practices indicated that WT filtering for GAT cannot provide a good de-noising effect with these decomposed levels [37]. (3) Several previous studies used computer-simulated signals to verify the effect of the filtering model, which lacked verification by actual observed data. Metrics such as signal-to-noise ratio, mean square error, smoothness and correlation coefficient are often used to evaluate the quality of the filtering model [35], while less attention has been paid to investigating the relationship between the variation of these metrics and the wavelet decomposed level.

To solve the above problems and improve the robustness and environmental adaptability of the GAT exposed to vibration, based on the WT and time-frequency analysis theory [11], a filter adapting to GAT data was designed. In our research the observed signals of the GAT were collected under the conditions of wind-induced vibration and ground vibration caused by vehicles passing. By comparing the difference between the filtered (and unfiltered) orientation results and the relative true north azimuth (high-precision Global Navigation Satellite System (GNSS) baseline established in the experimental area, with an orientation accuracy better than 0.5″), we determined the optimal wavelet decomposed level suitable for GAT data affected by different environmental factors, and a field experiment was carried out to verify the feasibility of our study.

The main structure of the manuscript is composed of four sections. Section 1 is an introduction to GAT and a literature review of the application of the WT filter. Section 2, Materials and Methods, includes three parts: Section 2.1, Data Characteristics of the GAT; Section 2.2, Basic Theory of WT; and Section 2.3, Experimental Design. Section 3 covers results and a discussion of the filtering experiment. Section 4 is the conclusion of our study.

## 2. Materials and Methods

### 2.1. Data Characteristics of the GAT

The observed signal collected by the GAT is the real-time discrete dynamic electro-optical parameter: the rotor current of the damping torque [43]. The gyro bearing can be calculated by the north-seeking algorithm from the electric signals, and there is a linear correlation between the gyro bearing and the rotor current [37]. According to the law of error propagation, the smaller the standard deviation of the rotor current, the higher the accuracy of the north-seeking result will be, which provides a theoretical possibility for improving the north-seeking accuracy of sensors by using a filtering model [10].

Differently from attitude gyros, which are susceptible to random drift even in an ideal working environment, the GAT adopts a magnetic suspension-supporting and photoelectric torque feedback technology to restrict the gyro precession, so that the gyro axis does not oscillate about the meridian direction but remains relatively static [37]. In the ideal state without external interference, the rotor current signal does not drift systematically, but always presents a stable overall trend accompanied by many random noises [10]. However, a non-stationary signal shows the effect of the disturbances of the external environment. Therefore, we have attempted to correct these abnormal signals using the WT filtering method to improve the orientation accuracy of the GAT.

### 2.2. Basic Theory of Wavelet Transform

The wavelet transform is defined as [12]:

$\psi (t)\in L^{2}(R)$, where $\overset{⏜}{\psi }(\omega )$ is the Fourier transformation of ψ(t), when $\overset{⏜}{\psi }(\omega )$ meets the follow conditions:

(1) $$C_{\psi }=\int_{R}\frac{{\left|\overset{⏜}{\psi }(\omega )\right|}^{2}}{\left|\omega \right|}d\omega <\infty .$$

ψ(t) is defined as a generating function, and the wavelet sequence $\psi_{a,b}(t)$ can be obtained as:

(2) $$\psi_{a,b}(t)=\frac{1}{\sqrt{\left|a\right|}}\psi (\frac{t-b}{a}),(a,b\in R;a\neq 0)$$

where a is the stretch factor and b is the translating factor. For an arbitrary function $f(t)\in L^{2}(R)$, the continuous wavelet transform can be expressed as:

(3) $$W_{f}(a,b)=<f,\psi_{a,b}>={\left|a\right|}^{-\frac{1}{2}}\int_{R}f(t)\psi (\frac{t-b}{a})dt.$$

**Figure 1:**
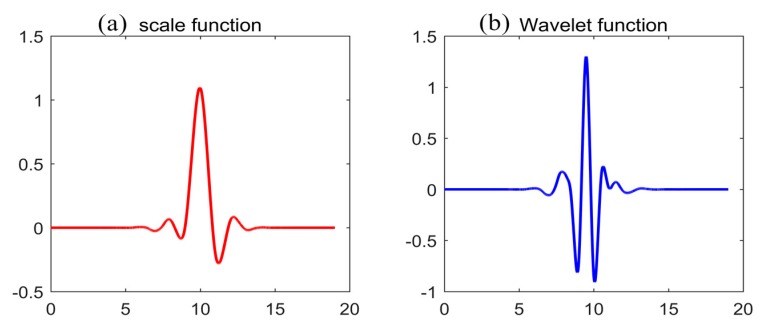
Scale function and wavelet function of Sym10.

The Mallat algorithm is used to decompose signals with orthogonal scaling functions and wavelet functions under two-scale coefficients [44]. The scale function constitutes a low-pass filter, while the wavelet function constitutes a high-pass filter. The signal is divided into low-frequency and high-frequency coefficients, and the low-frequency coefficients are decomposed layer by layer, so the final signal is decomposed into a residual low-frequency coefficient and a series of high-frequency coefficients [45]. The formula of the Mallat algorithm can be expressed as:

(4) $$\begin{array}{l} a_{j,k}=<f,\varphi_{j,k}>=\sum_{n\in Z}f(t)\varphi_{j,k}(t),k\in Z\\ d_{j,k}=<f,\psi_{j,k}>=\sum_{n\in Z}f(t)\psi_{j,k}(t),k\in Z \end{array}$$

where *f* is the original observed signal; $a_{j,k}$ and $d_{j,k}$ are decomposed low-frequency and high-frequency coefficients, respectively; and $\varphi_{j,k}$ and $\psi_{j,k}$ are the scale function and wavelet function, respectively. Symlets wavelet function is selected for our study, which can reduce the phase distortion in signal analysis and reconstruction and is suitable for the original signal processing of a north-seeking gyroscope in continuity, support length and filter length [40]. The number of vanishing moments for the Symlets wavelet function in our study is 10 (Sym10). The images of $\varphi_{j,k}$ and $\psi_{j,k}$ are shown in Figure 1.

#### 2.2.1. Determination of Threshold Function

The threshold de-noising is carried out for the high-frequency coefficients by corresponding criteria after wavelet decomposition. To smooth the contaminated signal, a soft threshold function is selected to process the signal of GAT. The criterion of the soft threshold function is [35]:

(5) $${\hat{d}}_{j,k}=\left\{ \begin{array}{ll} sign(d_{j,k})(\left|d_{j,k}\right|-\lambda ) & ,\left|d_{j,k}\right|\geq \lambda \\ 0 & ,\left|d_{j,k}\right|<\lambda \end{array}\right.$$

where $d_{j,k}$ is the high-frequency wavelet coefficients, λ is the threshold, and *sign*(*x*) is defined as:

(6) $$sign(x)=\left\{ \begin{array}{ll} -1, & x<0\\ 0, & x=0\\ +1, & x>0 \end{array}\right..$$

The Rigrsure adaptive threshold setting method is selected for our study, which can better preserve the smoothness and similarity of the signal [35]. The calculation process is as follows:

(7) $$\lambda_{k}=\sqrt{sx(k)}=\sqrt{\left(sort({\left|d_{j,k}\right|}^{2})\right)},\;k=0,1,2\ldots M-1$$

where *sx*(*k*) represents the sort command in program code. The risk curve generated by this threshold is:

(8) $$Rish(k)=\left[M-2k+\sum_{j=1}^{k}sx(j)+(M-k)sx(M-k)\right]/M,\;k=0,1,2\ldots ,M-1.$$

When the risk curve *Rish*(*k*) = min, the Rigrsure adaptive threshold can be defined as:

(9) $$\lambda =\sqrt{sx(k_{\min})}.$$

When λ is substituted into Equation (5), the modified high-frequency wavelet coefficients ${\hat{d}}_{j,k}$, after thresholding at each level, can be obtained.

#### 2.2.2. Reconstruction of Wavelet Coefficient and Time-Frequency Analysis

A Mallat inverse operation is performed on the low-frequency wavelet coefficient $a_{j,k}$, and the modified high-frequency coefficients ${\hat{d}}_{j,k}$ and on the coefficients after de-noising, and the filtered signal *F*(*t*) is reconstructed [44] as:

(10) $$F(t)=\sum_{n\in Z}a_{j,k}\varphi_{j,k}+\sum_{n\in Z}{\hat{d}}_{j,k}\psi_{j,k},k\in Z.$$

According to the north-seeking algorithm [10], the gyro bearings can be calculated from the reconstructed signals after wavelet de-noising. The Mallat algorithm obtains a series of wavelet coefficients with different frequencies, a time series is projected onto the time-scale plane, and the time and frequency components of the signal are obtained simultaneously [45]. The sampled signal is expressed as a 3D time-frequency spectrum of time, instantaneous frequency and wavelet coefficients.

For the scale parameter *a*, it is necessary to ensure that the frequency range of the Sym10 wavelet function is greater than or equal to that of the observed signal. According to the sampling theorem, the sampling frequency of the signal is at least twice the maximum frequency, and the conversion relationship between the scale parameter and the frequency of the wavelet function is as follows:

(11) $$F_{a}=F_{c}\times F_{s}/a$$

where *F_a_* is the frequency of the wavelet function; *F_S_* is the signal sampling frequency; and *Fc* is the central frequency of the wavelet function. For Sym10, *F_c_* = 0.6842 Hz. To make sure the frequency range of the wavelet function is (0, *F_S_*/2), the frequency scale range of the wavelet function should be (2*F_C_*, +∞); *a* = 100 for GAT data in our research.

#### 2.2.3. Determination of the Optimal Decomposed Level

The optimal decomposed level of a signal is generally determined by the root mean square error (RMSE), signal-to-noise ratio (SNR), correlation coefficient (ρ) and smoothness (r) of the signal [35]:

(12) $$\begin{array}{l} RMSE=\sqrt{\left[\sum_{i=1}^{n}{\left(f(i)-F(i)\right)}^{2}\right]/n}\\ SNR=10\mathrm{lg}(\sum_{i=1}^{n}{\left(f(i)\right)}^{2}/\sum_{i=1}^{n}{\left(f(i)-F(i)\right)}^{2})\\ \rho =\mathrm{cov}(F,f)/(\sigma_{F}.\sigma_{f})\\ r=\sum_{i=1}^{n-1}{\left(F(i+1)-F(i)\right)}^{2}/\sum_{i=1}^{n-1}{\left(f(i+1)-f(i)\right)}^{2} \end{array}$$

where *f(i)* and *F(i)* are the original signal and the reconstructed signal, respectively; $\sigma_{f}$ and $\sigma_{F}$ are the variance of the original signal and the reconstructed signal, respectively; and n is the signal length. A variable coefficient weighting method combines the above indexes to a composite index [46]. The above indexes of alternative reconstructed signals at different decomposed levels are normalized into [0–1]:

(13) $$\begin{array}{l} PRMSE=\frac{\max(RMSE)-RMSE}{\max(RMSE)-\min(RMSE)}\\ PSNR=\frac{SNR-\min(SNR)}{\max(SNR)-\min(SNR)}\\ P\rho =\frac{\rho -\min(\rho )}{\max(\rho )-\min(\rho )}\\ Pr=\frac{\max(r)-r}{\max(r)-\min(r)} \end{array}$$

Then the indexes are weighted by the coefficients of variation:

(14) $$\begin{array}{l} W_{PRMSE}=\frac{CV_{PRMSE}}{CV_{PRMSE}+CV_{PSNR}+CV_{P\rho }+CV_{Pr}}\\ W_{PSNR}=\frac{CV_{PSNR}}{CV_{PRMSE}+CV_{PSNR}+CV_{P\rho }+CV_{Pr}}\\ W_{P\rho }=\frac{CV_{P\rho }}{CV_{PRMSE}+CV_{PSNR}+CV_{P\rho }+CV_{Pr}}\\ W_{Pr}=\frac{CV_{Pr}}{CV_{PRMSE}+CV_{PSNR}+CV_{P\rho }+CV_{Pr}} \end{array}$$

where CV=σ/μ is the coefficient of variation of each index; *W* is the weight of each index in the composite index; and σ and μ are the variances and means of each index under different decomposed level conditions, respectively. The composite index *H* can be calculated by:

(15) $$H=PRMSE\times W_{PRMSE}+PSNR\times W_{PSNR}+P\rho \times W_{P\rho }+Pr\times W_{Pr}.$$

When *H* = max, the corresponding optimal decomposed level can be determined.

### 2.3. Experimental Design

**Figure 2:**
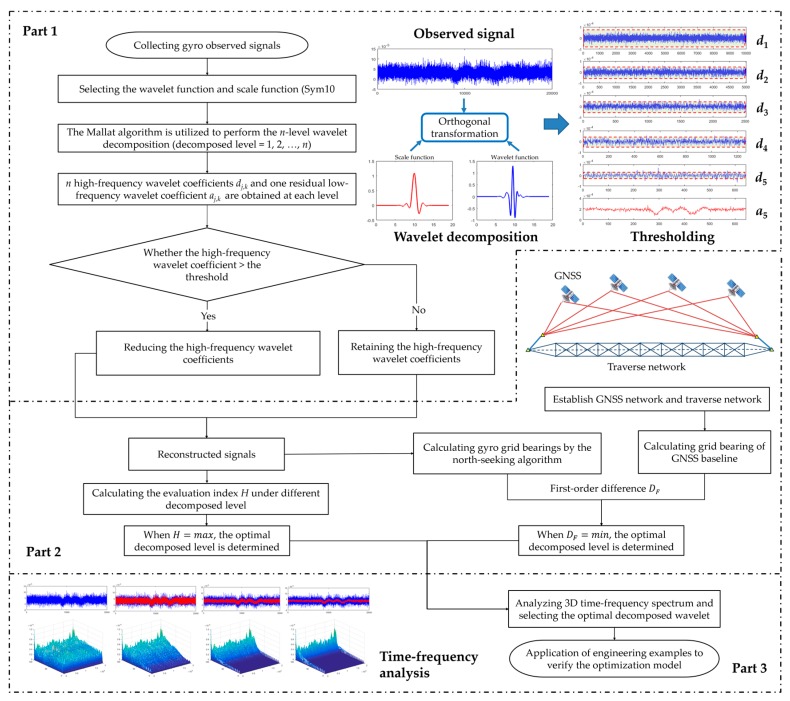
The flow chart of the experiment of the optimal wavelet decomposed level.

A field experiment was designed to explore the effect of different wavelet levels for de-noising on improving gyroscope data exposed to vibration (Figure 2). The experiment was divided into three parts: Part 1: wavelet decomposition and threshold; Part 2: optimization of the decomposed level; Part 3: spectrum analysis and feasibility verification. The details of the experimental design are as follows.

#### 2.3.1. Experimental Network

**Figure 3:**
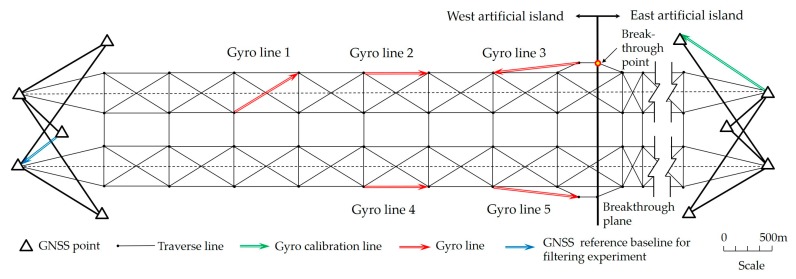
Design of the experimental network.

The experimental site (longitude: E 113.6°; latitude: N 22.1°) was located on a town road with a subtropical monsoon climate where it is often windy during summer nights. The road lies in an approximate east–west direction and has an occasional vehicle passing along it. More engineering details of the Hong Kong‒Zhuhai‒Macao Bridge can be found in References [10,47]. A GNSS network and traverse network with the same scale as the tunnel network were designed on the experimental site (Figure 3) [10]. The design of the experimental network is shown in Table 1 and the gyro observations in the experimental network are shown in Table 2.

All gyro grid bearings in the experiment needed to be corrected by the gyro calibration, which was measured on the gyro calibration line (green arrow in Figure 3) in a stable observation environment; the accuracy of the gyro bearing was better than 1.3″ (Table 2). The relative grid bearing accuracy of the selected GNSS baseline (0.5″) was much better than that of the observed gyro bearings exposed to vibration (6.2″). Therefore, the grid bearing of the GNSS baseline could be regarded as a relative true north azimuth for checking gyro grid bearings in our experiment.

Wind induced and physical vibrations are the most important environmental factors affecting the estimation of the orientation accuracy [10], and the experimental data were collected under the influence of these vibration factors. The influence of other errors in our experiments needed to be eliminated, such as centering error, lateral refraction error and temperature drift error of the gyro sensor [10,51]. Some measures were taken to reduce the impact of these errors: (1) all gyro measurements were carried out on stations with pillars and forced centering plates, therefore, the centering precision was better than 0.2 mm; (2) our experiment was carried out above-ground and the lateral refraction effects would have been very weak; and (3) the working interval of the gyro sensor was 20 min, so that the gyro motor with high speed rotation could be cooled.

#### 2.3.2. Optimization of the Decomposed Level

To determine the optimal decomposed level for GAT data, the GNSS reference baseline (blue arrow in Figure 3) with higher north-seeking orientation accuracy was considered a constraint condition. The gyro signals on the GNSS reference baseline for the filtering experiment are classified according to different waveform distributions caused by different environmental factors. Alternative reconstructed signals are generated for different types of observed signals with different decomposed levels. The gyro grid bearings of the reconstructed signals under different decomposed levels will be calculated respectively by the north-seeking algorithm [10]. According to Equation (11), the 3D time-frequency spectra of the reconstructed signals will be drawn to investigate the change rule of the optimal decomposed level. 

Two kinds of indexes were used to evaluate and compare the de-noising effect of filtering under different decomposed levels: (1) the normalized composite index *H* mentioned in Section 2.2.3; when *H* = max, the corresponding decomposed level is the optimum. (2) Define the absolute differences between the filtered gyro grid bearings and the GNSS grid bearings as *D_F_*, which represents the corrective effect of the filtering model for the north-seeking result. When *D_F_*= min, the optimal decomposed level can be determined. 

#### 2.3.3. Feasibility verification

To verify the feasibility of this method, three kinds of filtering schemes were adopted to process the observed gyro signals on the 5 gyro (traverse) lines in the network (red arrows in Figure 3): (i) unfiltered, the wavelet decomposed level = 0; (ii) filtering with all wavelet decomposed levels = 5, which is the empirical value in previous studies [41,42,43]; (iii) filtering with the optimal decomposed level determined by the above experiments. Two types of indicators were used to compare the different filtering schemes in our study: (1) define the absolute differences between the filtered gyro grid bearings and the classical survey bearings as *D_T_*, which represents the external coincidence accuracy of gyro bearings; (2) the lateral breakthrough error (LBE) at the breakthrough point (Figure 3), which represents the lateral coordinate difference value of the point between the western and eastern island networks with gyro observations [10]. The smaller the above indicators are, the better the obtained de-noising effect of the signal processing scheme will be.

## 3. Results and discussion

### 3.1. Optimization of the Decomposed Level

**Figure 4:**
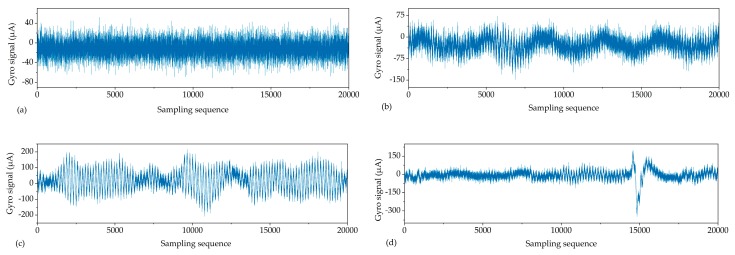
Time series of observed signals: (**a**) steady; (**b**) periodic; (**c**) jitter; (**d**) jumping.

**Figure 5:**
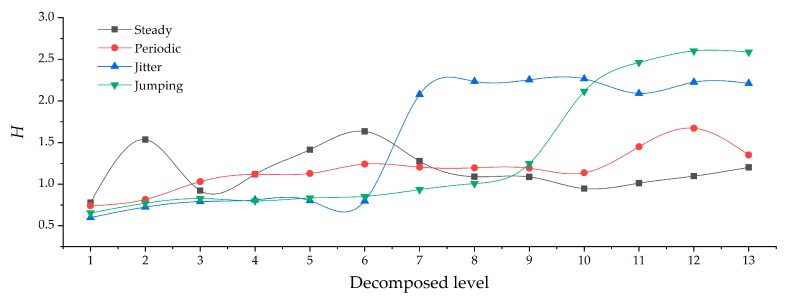
Comparison of H for different types of reconstructed signals under different decomposed levels.

**Figure 6:**
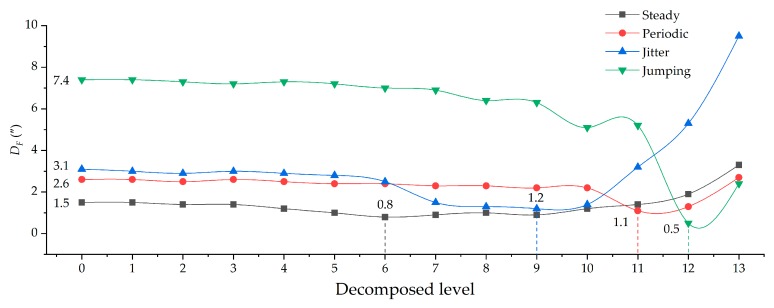
Comparison of *D_F_* for different types of reconstructed signals under different decomposed levels.

The observed signals on the GNSS reference baseline in our study can be categorized into four typical types: (a) steady signal; (b) periodic signal; (c) jitter signal; and (d) jumping signal (Figure 4). *H* and *D_F_* of the four types of reconstructed signals under different decomposed levels are shown in Figure 5 and Figure 6, respectively.

**Figure 7:**
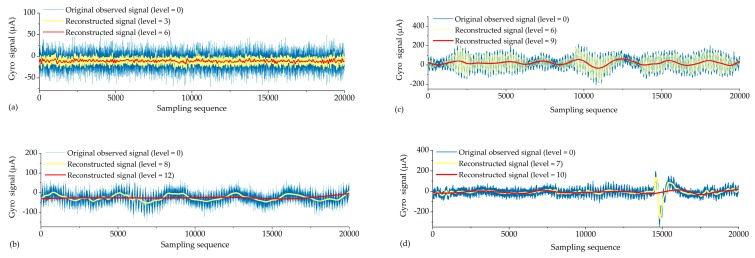
Time series of reconstructed signals: (**a**) steady; (**b**) periodic; (**c**) jitter; (**d**) jumping.

**Figure 8:**
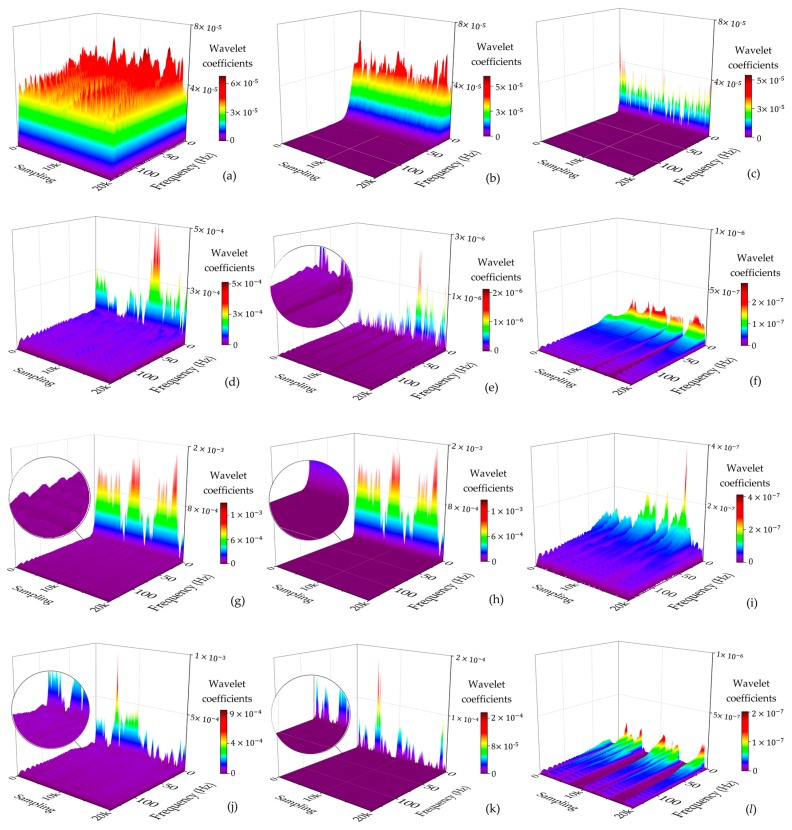
3D time-frequency spectra of four types of signals under different decomposed levels: (**a**) steady (level = 0); (**b**) steady (level = 3); (**c**) steady (level = 6); (**d**) periodic (level = 0); (**e**) periodic (level = 8); (**f**) periodic (level = 12); (**g**) jitter (level = 0); (**h**) jitter (level = 6); (**i**) jitter (level = 9); (**j**) jumping (level = 0); (**k**) jumping (level = 7); (**l**) jumping (level = 10).

From the overall comparison results after filtering, the accuracy of all types of reconstructed signals had been improved to some extent. The greater the *H*, the better the de-noising effect (Figure 5), while the smaller the *D_F_*, the better the corrective effect of the north-seeking results (Figure 6). With the increase of decomposed level, different types of observed signal have different optimal decomposed levels under the constraint of *H* = max or *D_F_* = min (Figure 5 and Figure 6). The time series of reconstructed signals (Figure 7) and their corresponding 3D time-frequency spectra (Figure 8) were drawn to analyze the changing rules of the four types of observed signal, respectively.

1. Steady signals were collected under ideal observation conditions. Since the observed signals were less affected by the external environment, *D_F_* was already relatively small before filtering (Figure 6). The distribution of wavelet coefficients on each frequency band is uniform, and the observed signal contains a significant amount of high frequency random noise (Figure 8a). By increasing the decomposed level from 0 to 6, most of the high-frequency wavelet coefficients had been removed gradually (Figure 8b,c), the reconstructed signal became smoother, and white noise was eliminated (Figure 7a), while the decomposed level had little effect on *D_F_* (from 1.5″ to 0.8″) (Figure 6). Consequently, the optimal decomposed level mainly depends on *H*. When the decomposed level = 6, *H* = max, and good results both on de-noising (Figure 7a) and corrective effect (Figure 6) were achieved.
2. Periodic signals were collected under the conditions where interference can be ignored. Like the pattern of steady signals, high-frequency noise accounts for most of the spectra, while the periodic low-frequency coefficient occupies most of the amplitude energy (Figure 8d). When the decomposed level = 8, most of the Gaussian noise was eliminated, but the time series of signals still retained periodic characteristics (yellow line in Figure 7b) since the low-frequency wavelet coefficients had a large residual in the spectra (Figure 8e). To remove the periodicity and make the signal smoother (red line in Figure 7b), the decomposed level needed to reach 12 (Figure 5 and Figure 6). Consequently, only very small wavelet coefficients (smaller than 2 × 10^−7^) were left in the spectrum (wavelet in Figure 8f) and *D_F_* changed from 2.6″ to 1.3″ (Figure 6). This periodic trend is the weak response to the environmental factors of the torque rectification algorithm of GAT, which belongs to regular systematic error [7]. It affects the signal dispersion (RMSE) but has less influence on the north-seeking result (mean value). Therefore, it is not necessary to completely eliminate the periodic trend in the signal.
3. Jitter signals were collected under wind-vibration conditions. Although the high-frequency wavelet coefficients occupied a wide frequency band range, low-frequency wavelet coefficients occupied a larger proportion of energy (Figure 8g), which led to the observed signal containing the jitter trends (Figure 7c). Most high-frequency random noise was removed at the decomposed level = 6. However, residual low-frequency wavelet coefficients remained (Figure 8h), indicating the noise affected by the jitter trend had not been eliminated (yellow line in Figure 7c) and that further decomposing and thresholding needed to be carried out. The comparison of the spectrum at level = 6 (Figure 8h) and level = 9 (Figure 8i) shows that residual wavelet coefficients decreased from 1 × 10^-−3^ to 2 × 10^−7^, H changed from 0.8 to 2.3 (Figure 5), *D_F_* changed from 2.4″ to 1.1″ (Figure 6), the reconstructed signal became smooth, and the abnormal signals affected by environmental factors were repaired (red line in Figure 7c). Continuing to increase the decomposed level, H gradually converged to a certain value and did not obviously change anymore (Figure 5). However, a negative effect on *D_F_* occurred when the decomposed level exceeded 10 (Figure 6), which indicates that an excessive decomposed level increases the risk of reconstructed signal distortion.
4. Jumping signals were collected under the condition of ground vibration caused by a vehicle passing. Most of the energy was concentrated in the low-frequency band, and the jumping trend formed a peak in the low-frequency band in the spectrum (Figure 8j). The energy of high-frequency noise seemed negligible compared with the peak, and almost all high-frequency noise was eliminated at the decomposed level = 7, while the energy of the peak remained in the spectrum (Figure 8k). The time series shows that the jumping trend had not been removed entirely (Figure 7d), and the slopes of *H* and *D_F_* were also not large (Figure 5 and Figure 6); *D_F_* changed from 7.4″ to 6.9″. With an increase in the decomposed level from 7 to 10, the slope of *H* and *D_F_* increased significantly (Figure 5 and Figure 6). The peak flattened (Figure 8l) and the jumping trend was corrected with a corrective effect of 1.3″ achieved (from 6.9″ to 5.6″) (Figure 6 and Figure 7d). After increasing the decomposed level from 10 to 12 (*H* = max, *D_F_* = min), a change of H was no longer obvious (Figure 5), while *D_F_* decreased dramatically (from 5.6″ to 0.5″) (Figure 6). Although this seems to be beneficial to the north-seeking results, it is not; drastic change is risky. Even though the result shows that the gyro grid bearing after filtering is closer to the true north, similar to the case of the jitter signals, an excessive decomposed level causes instability of *D_F_*, which reduces the reliability of north-seeking results.

In summary, wavelet filtering can repair the abnormal signals affected by the environment to a certain extent. This repair capability is finite, which means that it is impossible for *D_F_* to approach zero infinitely after filtering. This is because even after correcting for the errors caused by environmental factors, the observed signal may contain some system errors that we cannot eliminate. Fortunately, combined with the above analysis of *H* and *D_F_* results, we can determine the most suitable decomposed level corresponding to different types of environmental factors and optimize the de-noising and corrective effects of wavelet filters (Table 3).

For steady and periodic signals, which are less affected by the external environment, *H* and *D_F_* improved slowly with the increase of decomposed level, and a moderate decomposed level (6 to 8) can meet the requirements of the correction of the north-seeking result (Table 3). For jitter and jumping signals that are heavily disturbed by environmental factors, a higher decomposed level (8 to 10) should be carried out to further correct the abnormal trend caused by low-frequency disturbances (Table 3). However, this does not mean that the higher the decomposed level, the better the north-seeking result that will be achieved. Excessive filtering may cause negative effects and worsen the north-seeking result. Consequently, the wavelet decomposition should not exceed level 10 for the GAT data filtering. Thus, although periodic and jumping signals show better *H* and *D_F_* results at a higher decomposed level = 12, we still choose an optimal decomposed level = 8 or 10 (Table 3), which is safer and meets the de-noising requirements.

### 3.2. Feasibility Verification

**Figure 9:**
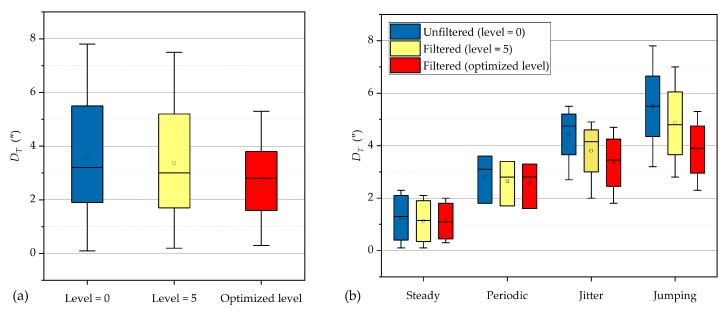
The boxplots of *D_T_*: (**a**) *D_T_* for different filtering schemes; (**b**) *D_T_* for each type of observed signal.

To verify the correctness of the optimal wavelet decomposed level, additional gyro observed signals were collected independently on five gyro lines (Table 2 and Figure 3), so that a total of 15 observed signals were classified according to the above environmental factors. Two types of precision indicators (*D_T_* and LBE) mentioned in Section 2.3.3 were used to compare the filtering effect in our study. The boxplots of *D_T_* for different filtering schemes and for each type of observed signal are shown in Figure 9a,b.

In Figure 9a, compared with the unfiltered signal, *D_T_* showed a significant difference compared to filtering with the optimal decomposed level (*p* < 0.01, *n* = 15). Compared to the filter with decomposed level = 5, *D_T_* also showed a significant difference to the filter with the optimal decomposed level (*p* < 0.01, *n* = 15), which suggested that the accuracy of the north-seeking results was improved compared to the empirical decomposed level in previous studies [40,41,42]. This shows the benefit of the optimization of the wavelet decomposed level according to different types of observed signals, so that the de-noising and corrective effect of north-seeking results can be improved as much as possible. In Figure 9b, the more obvious the change in *D_T_* is, the more significant the de-noising effect of the wavelet filter. The result showed that the de-noising effect on jitter and jumping signals is better than on steady and periodic signals, which is consistent with the result of the above experiments in Section 3.1. A significance test was made on *D_T_* with the three kinds of filtering scheme mentioned in Section 2.3.3: (1) unfiltered (decomposed level = 0); (2) filtering with decomposed level = 5; and (3) filtering with the optimal decomposed level.

**Figure 10:**
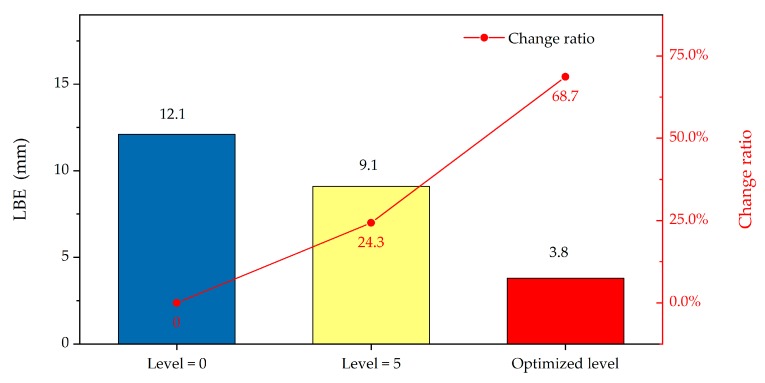
Comparison of lateral breakthrough error (LBE) for different filtering schemes.

The lateral breakthrough error (LBE) is a common precision indicator to check whether the gyro data can correct the grid bearing of the underground network in a tunnel. In our study, the LBE of the breakthrough point (Figure 3) was calculated by the combined adjustment of the underground control network with filtered gyro data. The result shows that the LBE achieved a 68.7% gain effect (from 12.1 mm to 3.8 mm) compared with unfiltered gyro data, which further verified the effectiveness and practicability of the proposed optimal filtering model (Figure 10).

In our study, the observations were obtained under the influence of known environmental factors, such as wind vibration or vehicle vibration. According to the natural environment, noise sources are uncertain in practical applications. Therefore, the optimal decomposition levels of wavelet filters are not classified according to the magnitude of the observed signal noise, but are mainly determined by the wave patterns of the observations because, although the magnitude of noise is different, the influence of similar error sources on the observations has common characteristics.

Therefore, if we do not know clearly which environmental factors affect the observations in the data acquisition process, we can select the appropriate filter decomposition level from Table 3 according to the wave pattern of the time series. Conservatively, even if we do not know the wave characteristics of the observation, according to the experimental results it is relatively safe to select any level from Level 6 to Level 10 to potentially improve the north-seeking accuracy.

## 4. Conclusions

From the above experimental results, the following conclusions can be drawn:

1. The observed signal of a north-seeking gyro sensor is affected by different environmental factors and shows different non-stationary characteristics, so that the selection of the wavelet decomposed level for de-noising is adapted to different types of observed signals.
2. Using the constraints of high-precision external verification conditions (GNSS grid bearings) combined with the prior observed signals will provide empirical values for the wavelet decomposed level and optimize the efficiency of the filtering model. This method of determining the optimal wavelet decomposed level is able to be used not only in GAT, but also in other types of gyroscope sensors.
3. In the application of tunnel surveying engineering, which is vulnerable to complex environmental factors, the optimized model eliminates the influence of external disturbances on the observed signal to a certain extent and enhances the north-seeking accuracy of gyro sensors.

Due to the limitation of the length of the article and experimental data, we only classified the observed signals into four typical categories. The classification of observed signals under different environmental impacts needs to be further examined by more sophisticated experiments. To further improve the de-noising effect, different thresholding methods will be adopted for high-frequency coefficients of different decomposed levels in our future research. Another filtering method suitable for non-stationary GAT signal analysis, the Hilbert–Huang transform, will also be discussed and compared with the wavelet method in subsequent research.

## Figures and Tables

**Table 1:** Design of the experimental network.

Type of Network	Number of Observations	Instrument	Precision Indexes	Standard Deviation of the Weakest Points
Global Navigation Satellite System (GNSS) network	10 points simultaneously observed by GPS for 76 hours	Trimble R7 GPS receiver [48]	Static plane accuracy ± (3 mm + 0.1 ppm)	1.2 mm (Use GLOBK Software for 3D network adjustment [49])
Traverse network	Horizontal directions: 221 (9 repeat observations per stations) Horizontal distances: 119 (9 repeat observations per stations)	Leica TS30 total station [50]	Direction precision ± 0.5″ Distance precision ± (0.6 mm + 1 ppm)	1.0 mm (Use COSA-CODAPS Software for network adjustment)

**Table 2:** Summary of the gyro observations in the experimental network.

Type of Survey Lines	Parameters of Survey Lines		Experimental Parameters of Gyro Observation		
	Length	Relative Accuracy of Grid Bearing	Repeat Observation Per Station	Observation Environment	Standard Deviation of Gyro Bearing
Gyro calibration line (GNSS baseline)	613 m	0.4″	4	Stable	1.3″
GNSS reference baseline for filtering experiment	524 m	0.5″	4	Stable, vehicle passing or wind vibration	6.2″
Gyro line 1	744 m	1.1″	3	Wind vibration and vehicle passing	2.3″
Gyro line 2	696 m	1.3″	3	Vehicle passing	1.5″
Gyro line 3	319 m	1.8″	3	Stable	1.3″
Gyro line 4	692 m	1.3″	3	Weak wind vibration	1.8″
Gyro line 5	244 m	1.9″	3	Stable	2.0″

Sampling frequency of gyro sensor: 20,000 in 75 seconds. Nominal accuracy of gyro orientation: 3.5″, the drift of gyroscope calibration is about 10″/year.

**Table 3:** Optimal wavelet decomposed level of different types of observed signal.

Criteria	Steady	Periodic	Jitter	Jumping
*H* = max	6	12	10	12
*D_F_* = min	6	11	9	12
Comprehensive evaluation	6	8	9	10

## References

[1] Bai J.M., Zhao G.S., Rong H.J., Wang X.H. (2018). Seeker-Azimuth Determination with Gyro Rotor and Optoelectronic Sensors. Sensors.

[2] Lauf G.B. (1963). The Gyrotheodolite and Its Application in The Industry of South Africa. J. S. Afr. Inst. Min. Metall..

[3] Velasco-Gómez J., Prieto J.F., Molina I., Herrero -Tejedor T.R., Fábrega J., Pérez-Martín E. (2016). Use of the Gyrotheodolite in Underground Networks of Long High-speed Railway Tunnels. Surv. Rev..

[4] Li K., Wang L., Lv Y.H., Gao P.Y., Song T.X. (2015). Research on the Rapid and Accurate Positioning and Orientation Approach for Land Missile-Launching Vehicle. Sensors.

[5] Shi Z., Yang Z.Q., Zhang Z. (2013). Study on Automatic North-Seeking Key Technologies of Maglev Gyroscope. Open Mech. Eng. J..

[6] Jia Z.Y., Ma X., Liu W., Lu W.B., Li X., Chen L., Wang Z.Q., Cui X.C. (2014). Pose Measurement Method and Experiments for High-speed Rolling Targets in a Wind Tunnel. Sensors.

[7] Yan Q.X., Li B.J., Zhang Y.Y., Yan J., Zhang C. (2017). Numerical Investigation of Heat-Insulating Layers in A Cold Region Tunnel:Taking into Account Airflow and Heat Transfer. Appl. Sci..

[8] Narayananellore S.K., Doko N., Matsuo N., Saito H., Yuasa S. (2017). Effect of MgO Underlying Layer on the Growth of GaOx Tunnel Barrier in Epitaxial Fe/GaOx/(MgO)/Fe Magnetic Tunnel Junction Structure. Sensors.

[9] Yi X.F., Zhang J., Fan T.H., Tian B.F., Jiang C.D. (2018). Design of Meter-Scale Antenna and Signal Detection System for Underground Magnetic Resonance Sounding in Mines. Sensors.

[10] Ma J., Yang Z.Q., Shi Z., Liu C.C., Yin H.Q., Zhang X.Z. (2019). Adjustment Options for A Survey Network with Magnetic Levitation Gyro Data in An Immersed Under-Sea Tunnel. Surv. Rev..

[11] Combes J.M., Grossmann A., Tchamitchian P. (1989). Wavelet: Time-Frequency Methods and Phase Space. Inverse Probl. Theor. Imaging.

[12] Dabuechies I. (2002). The Wavelet Transform, Time-Frequency Localization and Signal Analysis. IEEE Trans. Inf. Theor..

[13] Daubechies I., Sweldens W. (1998). Factoring Wavelet Transforms into Lifting Steps. J. Fourier Anal. Appl..

[14] Sweldens W. Lifting Scheme: A New Philosophy in Biorthogonal Wavelet Constructions. Proceedings of the Wavelet Applications in Signal and Image Processing III.

[15] Boles W.W., Boashash B. (2002). A Human Identification Technique Using Images of the Iris and Wavelet Transform. IEEE Trans. Signal Process..

[16] Gradolewski D., Magenes G., Johansson S., Kulesza W. (2019). A Wavelet Transform-Based Neural Network Denoising Algorithm for Mobile Phonocardiography. Sensors.

[17] Kutlu H., Avcı E. (2019). A Novel Method for Classifying Liver and Brain Tumors Using Convolutional Neural Networks, Discrete Wavelet Transform and Long Short-Term Memory Networks. Sensors.

[18] Grinsted A., Moore J.C., Jevrejeva S. (2004). Application of The Cross Wavelet Transform and Wavelet Coherence to Geophysical Time Series. Nonlinear Process. Geophys..

[19] To A.C., Moore J.R., Glaser S.D. (2009). Wavelet Denoising Techniques with Applications to Experimental Geophysical Data. Signal Process..

[20] Chang S.G., Yu B., Vetterli M. (2000). Adaptive Wavelet Thresholding for Image Denoising and Compression. IEEE Trans. Image Process..

[21] Antonini M., Barlaud M., Mathieu P., Daubechies I. (1992). Image Coding Using Wavelet Transform. IEEE Trans. Image Process..

[22] Yang Y., Tong S., Huang S.Y., Lin P. (2014). Dual-Tree Complex Wavelet Transform and Image Block Residual-Based Multi-Focus Image Fusion in Visual Sensor Networks. Sensors.

[23] Khan Z., Balch T., Dellaert F. (2004). An MCMC-Based Particle Filter for Tracking Multiple Interacting Targets. Comput. Vis.-ECCV.

[24] Chang C., Ansari R. (2005). Kernel Particle Filter for Visual Tracking. IEEE Signal Process. Lett..

[25] Breitenstein M.D., Reichlin F., Leibe B., Koller-Meier E., Gool L.V. Robust Tracking-by-Detection Using A Detector Confidence Particle Filter. Proceedings of the 2009 IEEE 12th International Conference on Computer Vision.

[26] Molina-Tenorio Y., Prieto-Guerrero A., Aguilar-Gonzalez R. (2019). A Novel Multiband Spectrum Sensing Method Based on Wavelets and The Higuchi Fractal Dimension. Sensors.

[27] Selim H., Prieto M.D., Trull J., Romeral L., Cojocaru C. (2019). Laser Ultrasound inspection Based on Wavelet Transform and Data Clustering for Defect Estimation in Metallic Samples. Sensors.

[28] Sabat S.L., Giribabu N., Nayak J., Krishnaprasad K. Characterization of Fiber Optics Gyro and Noise Compensation Using Discrete Wavelet Transform. Proceedings of the 2009 Second International Conference on Emerging Trends in Engineering & Technology.

[29] Li J.L., Xu H.L., He Q. (2010). Research and Improvement of Denoising Method of Fiber Optic Gyroscope Based on Wavelet Packet Analysis. Acta Opt. Sin..

[30] Mao B., Wei W.J., Tong W.J., Mei Z.X. MEMS Gyro Denoising Based on Second Generation Wavelet Transform. Proceedings of the 2010 1st International Conference on Pervasive Computing.

[31] Liu F.Q., Liu F.M., Wang W.J., Xu B. MEMS Gyro’s Output Signal De-Noising Based on Wavelet Analysis. Proceedings of the 2007 International Conference on Mechatronics and Automation.

[32] Lee W., Park C.G. (2014). Double Fault Detection of Cone-Shaped Redundant IMUs Using Wavelet Transformation and EPSA. Sensors.

[33] Yi T.H., Li H.N., Zhao X.Y. (2012). Noise Smoothing for Structural Vibration Test Signals Using an Improved Wavelet Thresholding Technique. Sensors.

[34] Ayrulu-Erdem B., Barshan B. (2011). Leg Motion Classification with Artificial Neural Networks Using Wavelet-Based Features of Gyroscope Signals. Sensors.

[35] Liu H., Wang W.D., Xiang C.L., Han L.J., Nie H.Z. (2018). A De-Noising Method Using The Improved Wavelet Threshold Function Based on Noise Variance Estimation. Mech. Syst. Signal Process..

[36] Yang Z.Q., Gong Y., Shi Z., Yun L. (2011). Change Monitoring of Earth Rotation Parameter with Maglev Gyroscope Precessional Torque. Trans. Nonferrous Met. Soc. China.

[37] Yang Z.Q., Shi Z., Yang J.H. (2017). North Seeking Principle and Measurement Application of Magnetically Suspended Gyroscope.

[38] Cheng G.D. (2005). A Roadbed Cooling Approach for The Construction of Qinghai–Tibet Railway. Cold Reg. Sci. Technol..

[39] Ma J., Yang Z.Q., Ji G.F., Sun J., Liu J., Yang Y., Fan S., Yu W. (2017). The Determination of Plumb-Line Deviation by Adopting GNSS/Leveling Method in Super Long Tunnel Break-Through Measurement. China Satellite Navigation Conference (CSNC) 2017 Proceedings: Volume I.

[40] Miao L.J. (2000). Application of wavelet analysis in the signal processing of the fiber optic gyro. J. Astronaut.

[41] Ren C.H., Xiong L.X., Zhao X.J., Pan Y.J. (2010). The Application of Wavelet Threshold—Value Filter in Signal Processing of Fiber Optic Gyroscope. Piezoelectrics Acoustooptics.

[42] Li Q., Teng J.F., Wang X., Zhang Y.Q., Guo J.C. Research of Gyro Signal De-Noising with Stationary Wavelets Transform. Proceedings of the IEEE Ccece Canadian Conference on Electrical & Computer Engineering.

[43] Ma J., Shi Z., Yang Z.Q. Variable precision adjustment optimization of a long traverse with Maglev gyro observations. Proceedings of the 16th International Congress for Mine Surveying.

[44] Mallat S.G. (1989). Multiresolution Approximations and Wavelet Orthonormal Bases of L 2 (R). Trans. Am. Math. Soc..

[45] Mallat S. (2008). A Wavelet Tour of Signal Processing: The Sparse Way.

[46] Dellwo V., Karnowski P., Szigeti I. (2006). Rhythm and Speech Rate: A Variation Coefficient for C. Language and Language-Processing.

[47] Huang S.X., Li G.Q., Wang X.P., Zhang W. (2017). Geodetic Network Design and Data Processing for Hong Kong–Zhuhai–Macau Link Immersed Tunnel. Surv. Rev..

[48] Trimble User Guide for Trimble R7 GPS Receiver. http://www.terraseis.com/ckfinder/userfiles/files/Trimble%20R7GNSS.pdf.

[49] Herring T.A., Floyd M.A., King R.W., McClusky S.C. GLOBK Reference Manul Global Kalman filter VLBI and GPS analysis program Release 10.6.

[50] Leica Geosystems Leica TPS1200+TS30/TM30 Technical Reference Manual.

[51] Johnston A. (1991). Lateral Refraction in Tunnels. Surv. Rev..

